# Landiolol hydrochloride for prevention of atrial fibrillation during esophagectomy: a randomized controlled trial

**DOI:** 10.1186/s40981-020-00338-3

**Published:** 2020-05-11

**Authors:** Yoshitaka Aoki, Yohei Kawasaki, Kazuki Ide, Yuichiro Shimizu, Shinsuke Sato, Junichiro Yokoyama

**Affiliations:** 1 Department of Anesthesiology and Intensive Care Medicine, Hamamatsu University School of Medicine, 1-20-1 Handayama, Higashi-Ku, Hamamatsu-shi, Shizuoka, 431-3192 Japan; 2 Department of Anesthesiology, Shizuoka General Hospital, Shizuoka, Japan; 3 Biostatistics Section, Clinical Research Centre, Chiba University Hospital, Chiba, Japan; 4 Faculty of Nursing, Japanese Red Cross College of Nursing, Tokyo, Japan; 5 Uehiro Research Division for iPS Cell Ethics, Center for iPS Cell Research and Application, Kyoto University, Kyoto, Japan; 6 Department of Gastroenterological Surgery, Shizuoka General Hospital, Shizuoka, Japan

**Keywords:** Landiolol hydrochloride, Atrial fibrillation, Esophagectomy, Intraoperative complications

## Abstract

**Introduction:**

Landiolol hydrochloride reduces the incidence of perioperative atrial fibrillation (AF) in cardiac surgery; however, little evidence is available regarding its effects in other types of surgery, including esophagectomy. We assessed the hypothesis that landiolol reduces perioperative AF and other complications associated with esophagectomy.

**Methods:**

This single-center, randomized, double-blind, parallel-group study enrolled patients scheduled for esophagectomy. Patients were divided into those given landiolol at 3 μg/kg/min or placebo for 24 h. The primary outcome was the proportion of patients who developed AF within 96 h starting at 9:00 am on the day of surgery. The secondary outcomes were the proportion of patients whose AF appeared within 24 h, other complications based on the Clavien–Dindo classification, and the intensive care unit and hospital stays.

**Results:**

Despite early study termination, 80 patients were screened, and 56 were enrolled (28/group) from September 2016 to June 2018. AF occurred within 96 h of surgery in six (21.4%) patients in the landiolol group and five (17.9%) patients in the placebo group (odds ratio, 1.26; 95% confidence interval, 0.33–4.7) and within 24 h of surgery in three (10.7%) patients in the landiolol group and two (7.1%) patients in the placebo group. There were no significant differences in the incidence of complications or in the number of intensive care unit or hospital stays between the groups.

**Conclusion:**

Although our small sample size prevents definitive conclusions, landiolol might not reduce the occurrence of AF or other complications.

**Trial registration:**

UMIN, UMIN000024040. Registered 13 September 2016, http://www.umin.ac.jp/ctr/index/htm

## Introduction

Atrial fibrillation (AF) is the most common arrhythmia during and after thoracic surgery, including esophagectomy [1]. The occurrence of postoperative AF is associated with an increased risk of pulmonary and anastomotic complications, resulting in an extended duration of hospital stay and increased mortality after esophagectomy [2–4]. The guidelines of the American Association for Thoracic Surgery suggest that some prophylactic drugs reduce the occurrence of postoperative AF [1]. However, some drugs are not widely available to patients because of adverse effects, and the optimal prophylactic therapy remains to be established.

Landiolol hydrochloride is an ultrashort-acting β1-selective blocker with a short half-life and does not trigger adverse events [5]. Several studies have shown that low-dose (0.5–10.0 μg/kg/min) landiolol administration prevents perioperative AF following cardiac surgery [6–10]. In contrast, only two randomized trials involved the administration of landiolol during esophagectomy [11, 12]. Landiolol administration has been shown to reduce the incidence of other complications following esophagectomy [11], an effect that has not been reported in cardiac surgery [7]. This finding must be interpreted with caution, however, because of limitations in the study protocol. Although the AF-preventive effect of landiolol has been studied extensively in cardiac surgery, there is little evidence regarding the effectiveness of its use in other types of surgery, including esophagectomy [5].

We hypothesized that low-dose landiolol can reduce the occurrence of perioperative AF and other complications during and after esophagectomy. The present study was performed to determine the preventive effect of landiolol compared with placebo in a double-blind randomized controlled trial with fully concealed allocation in patients undergoing scheduled esophagectomy.

## Patients and methods

The ethics board of Shizuoka General Hospital approved this study (SGHIRB #2015055). All procedures were performed in accordance with the Declaration of Helsinki, and written informed consent was obtained from each patient before enrollment. Before patient enrollment began, this trial was registered at the University Hospital Medical Information Network (Registration number: UMIN000024040).

In this prospective, single-center, two-arm, parallel-group, double-blinded randomized controlled trial, patients were randomly assigned to one of two parallel arms to receive either intravenous landiolol (intervention group) or normal saline solution (control group) during esophagectomy, each of which was continued for 24 h. The trial was conducted from September 2016 to June 2018 at Shizuoka General Hospital (Shizuoka, Japan). The trial is reported based on the Consolidated Standards of Reporting Trials (CONSORT) statement [13]. The trial was monitored by an independent clinical trial coordinator and evaluated by an independent data and safety monitoring committee to check for severe complications.

### Study participants

Participants undergoing scheduled esophagectomy with retrosternal reconstruction using a stomach conduit were eligible if they were 20 to 80 years old and deemed medically fit for surgery. The exclusion criteria were as follows: (1) history of AF, (2) history of second- or third-degree atrioventricular block, (3) preoperative bradycardia (heart rate of ≤ 50 beats/min), (4) abnormal thyroid function, (5) preoperative β-blocker use, (6) history of asthma, (7) hospitalization due to heart failure, (8) low heart function seen on preoperative echocardiography (left ventricular ejection fraction of < 30%), and (9) untreated pheochromocytoma.

### Intervention and control

The intervention was the administration of landiolol hydrochloride (Onoact®; Ono Pharmaceutical, Osaka, Japan) at 3 μg/kg/min for 24 h. Following establishment of general anesthesia, a central venous catheter was inserted for landiolol administration. Patients in the control group received normal saline solution (placebo) at the same start time and administration rate for 24 h.

### Outcome measures

The primary outcome was the proportion of patients who developed AF within 96 h (AF96h) beginning at 9:00 am on the day of surgery. For immediate detection of AF, patients underwent continuous electrocardiography for 96 h in the operating room, intensive care unit (ICU), and general ward. The diagnosis of AF was based on the absence of a P wave on the electrocardiogram for > 5 min. The anesthesiologist was responsible for the diagnosis of AF during surgery, and the surgeon was responsible after completion of surgery. AF was confirmed on a printed electrocardiogram with at least two physicians in agreement.

The secondary endpoints were the proportion of patients who developed AF within the first 24 h (AF24h) of surgery, the proportion of patients who developed other complications during hospitalization, and the length of the ICU and hospital stays. Complications were assessed by an esophageal surgical specialist using the Clavien–Dindo classification, which ranges from grade 0 (no complications) to grade V (death) [14, 15]. Clavien grade ≥ II complications were judged as clinically significant complications, and Clavien grade ≥ III complications were judged as serious complications. The follow-up period continued until hospital discharge.

### Randomization, blinding, and allocation concealment

An independent team allocated eligible patients to one of the two arms using a secure, computer-generated randomization list in a 1:1 ratio. The computer-generated randomization list was strictly controlled by an independent office that maintained allocation concealment until all patients had completed the study. The group assignment results were communicated to the pharmacy that prepared the clinical trial medications (i.e., landiolol and saline). Because the landiolol concentration was diluted based on body weight, the same volume of solution and the same administration rate were used in all patients. All staff members (surgeons, anesthesiologists, and nurses) remained blinded to the allocation until all patients had completed the study. Because all statistical analyses, including calculation of the sample size, were performed by external biostatisticians, allocation remained concealed for the data analysts.

### Anesthesia

Each patient entered the operating room at 9:00 am, which was the reference starting point for AF96h and AF24h. Standard monitoring in the operating room was performed by continuous five-lead electrocardiography, noninvasive blood pressure measurement, and pulse oximetry. Peripheral intravenous access via a forearm vein was established. Epidural anesthesia was performed in the lower thoracic region. General anesthesia was induced with propofol, which was administered by target-controlled infusion (TCI) of 4 μg/mL or as a bolus of 1.0 to 1.5 mg/kg, and remifentanil (0.4 μg/kg/min) followed by rocuronium (0.6–0.9 mg/kg) and tracheal intubation. A bronchial blocker was inserted for differential lung ventilation and adjusted appropriately using a bronchoscope. An indwelling catheter was then inserted into the radial artery. The central venous catheter was inserted from the right femoral vein using an echo-guided procedure. Immediately, landiolol or saline was administered through the central venous line using a syringe pump.

Anesthesia was maintained using propofol TCI or desflurane inhalation, depending on the anesthesiologist’s preference. In the propofol group, anesthesia was maintained with TCI at 2 to 4 μg/mL based on bispectral index monitoring. In the inhalation group, desflurane was applied to an end-tidal concentration of 4% to 6%. Analgesia was achieved with 4- to 7-mL boluses of 0.25% ropivacaine hydrochloride hydrate and continuous infusion of remifentanil up to 0.4 μg/kg/min. Rocuronium was administered intermittently based on the train-of-four ratio. The mean target blood pressure was 65 mmHg, and catecholamine was used at the discretion of the anesthesiologist.

After completion of the surgery, sugammadex was administered to antagonize any remaining neuromuscular blockade, and the patient was extubated in the operating room. All patients were then admitted to the ICU. The trial drug (landiolol or saline) was discontinued after 24 h. In the ICU, patients underwent infusion, transfusion, and/or administration of a catecholamine at the surgeon’s discretion. In accordance with the clinical path of our hospital, the ICU discharge criterion was a stable respiratory, circulatory, and metabolic status.

### Sample size calculation

Based on a previous report that AF occurs in approximately 20% of patients within 4 days after surgery [1], the incidence of AF was projected to be 5% in the landiolol group and 20% in the placebo group within 96 h. The study biostatistician independently estimated that a sample size of 188 patients (94 per group) was required to provide 80% power to detect a proportion difference of 15% between groups for the primary endpoint and a two-sided alpha level of 0.05 based on Fisher’s exact test.

### Statistical analysis

Continuous data are presented as mean and standard error or median (interquartile range), and categorical data are presented as number and percentage. For the primary outcome (AF96h), we calculated the overall proportion of AF96h occurrences and the proportion in the full analysis set (FAS), which was defined as all randomly assigned patients who received at least one dose of a study drug. The per-protocol set (PPS) was defined as the set after excluding patients who deviated from the protocol (e.g., surgical changes resulting from findings during surgery, additional landiolol, or administration of other beta-blockers). Differences between groups were evaluated using the risk difference and its 95% confidence interval (CI) and Fisher’s exact test. Additionally, odds ratios (ORs) and their 95% CIs were calculated using a logistic regression model. The adaptive lasso method was utilized for the sensitivity analysis [16]. Secondary outcomes of count data were evaluated using the same analytical method as for the primary outcome in the FAS. For the secondary outcomes of continuous data, the mean and standard error, median and interquartile range, and 95% CI of the mean difference were calculated. Student’s *t* test was used for comparisons between the two groups. A *P* value of < 0.05 was considered to indicate statistical significance in all analyses. All statistical procedures were performed using SAS version 9.4 for Windows (SAS Institute, Cary, NC, USA).

## Results

**Fig. 1:**
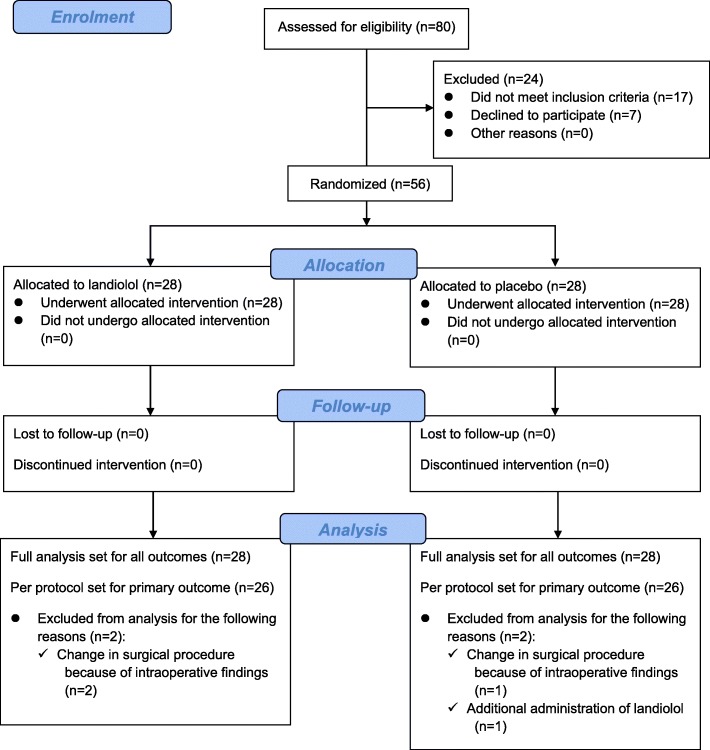
CONSORT flow diagram of the study

In April 2018, the Japanese Clinical Research Law was amended, making our research unsustainable because it did not meet the legal requirements. Thus, 80 patients who had been diagnosed with esophageal cancer and underwent esophagectomy from September 2016 to June 2018 were initially included in the study. Among these patients, 24 were excluded because the scheduled surgery was not a retrosternal reconstruction by stomach conduit (*n* = 10); the patient had a history of AF (*n* = 5), abnormal thyroid function (*n* = 1), or administration of a preoperative oral β-blocker (*n* = 1); or the patient refused to participate (*n* = 7). After inclusion in the trial, four patients did not follow the protocol. In three of these patients (two in the landiolol group, one in the placebo group), the surgical procedure was altered because of intraoperative findings by the surgeon. The fourth patient (placebo group) was administered landiolol because of postoperative tachycardia. We included these patients in the FAS based on the intention-to-treat principle. In addition, PPS analysis was performed for the primary outcome (Fig. 1). The baseline characteristics of the included patients and surgical data are shown in Table 1. Low-dose landiolol administration was not associated with any hemodynamic changes (Additional File 1), including the incidence of hypotension and bradycardia, during the study period.

**Table 1:** Baseline characteristics and surgical data for patients in the landiolol and placebo groups

**Characteristic**	**Landiolol**	**Placebo**	***P*value**
	**(*n*= 28)**	**(*n*= 28)**	
Age, years	68 (62–74)	69 (60–71)	0.36
Male sex	20 (71.4)	22 (78.6)	0.76
BMI, kg/m^2^	21 (20–23)	22 (20–23)	0.41
Ejection fraction, %	65 (60–69)	63 (61–66)	0.42
FEV_1.0%_, %	75 (73–79)	76 (71–80)	0.47
eGFR, mL/min/1.73 m^2^	62 (53–73)	66 (60–72)	0.49
**ASA class**			
I	5 (17.9)	8 (28.6)	0.36
II	21 (75.0)	20 (71.4)	
III	2 (7.1)	0 (0.0)	
**Comorbidities**			
Diabetes mellitus	5 (17.9)	2 (7.1)	0.42
Hypertension	15 (53.6)	6 (21.4)	0.026
Hyperlipidemia	6 (21.4)	1 (3.6)	0.10
COPD	0 (0.0)	1 (3.6)	1.0
CKD	12 (42.9)	7 (25.0)	0.26
Dialysis	0 (0.0)	0 (0.0)	1.0
Cerebrovascular disease	2 (7.1)	0 (0.0)	0.49
**Surgical data**			
Surgery time, min	442 (386–500)	429 (402–474)	0.93
Anesthesia time, min	512 (454–568)	501 (469–550)	0.86
Anesthesia method, TIVA	17 (60.7)	21 (75.0)	0.39
Catecholamine use	26 (92.9)	24 (85.7)	0.67
Infusion volume, mL	3765 (3125–4875)	3725 (3300–4260)	0.90
Transfusion volume, mL	315 (100–400)	340 (38–400)	0.90
Urine volume, mL	1175 (668–1456)	965 (764–1659)	0.58
Blood loss, mL	220 (144–476)	160 (120–303)	0.059

Data are expressed as number (%) or median (IQR)

*BMI* body mass index, *IQR* interquartile range, *ASA* American Society of Anesthesiologists, *COPD* chronic obstructive pulmonary disease, *FEV*_*1.0%*_ forced expiratory volume % in 1 s, *CKD* chronic kidney disease, *eGFR* estimated glomerular filtration rate, *TIVA* total intravenous anesthesia

All outcomes are summarized in Table 2. AF96h was reported in 11 patients. Among them and for the FAS, AF96h occurred in six (21.4% [95% CI, 6.2–36.6]) patients in the landiolol group and five (17.9% [95% CI, 3.7–32.0]) patients in the placebo group (risk difference, 3.6% [95% CI, − 17.2 to 24.4]; OR, 1.26 [95% CI, 0.33–4.7]). In the PPS, AF96h occurred in five (19.2% [95% CI, 4.1–34.4]) patients in the landiolol group and four (15.4% [95% CI, 1.5–29.3]) patients in the placebo group (risk difference, 3.9% [95% CI, − 16.7 to 24.4]; OR, 1.31 [95% CI, 0.31–5.55]). AF96h was not significantly different between the FAS (*P* = 1.00) and PPS (*P* = 1.00) groups. In a sensitivity analysis, the adaptive lasso method demonstrated that neither landiolol nor placebo allocation was associated with the occurrence of AF (Additional File 2).

**Table 2:** Primary and secondary outcomes

	Group (***n*** = 28 per group)		Risk difference, mean (95% CI)	P*
Parameter	Landiolol, ***n*** (%)	Placebo, ***n*** (%)		
Primary outcome				
AF96h in FAS	6 (21.4)	5 (17.9)	3.6 (− 17.2 to 24.4)	1.00
AF96h in PPS	5 (19.2)	4 (15.4)	3.9 (− 16.7 to 24.4)	1.00
Secondary outcomes				
Count data				
AF24h	3 (10.7)	2 (7.1)	3.6 (− 11.3 to 18.4)	1.00
Clavien grade ≥ II	11 (39.3)	14 (50.0)	− 10.7 (− 36.6 to 15.7)	0.59
Clavien grade ≥ III	7 (25.0)	7 (25.0)	0.0 (− 22.7 to 22.7)	1.00
Continuous data, days	Mean ± SE	Mean ± SE	Mean difference (95% CI)	*t* test
	Median (IQR)	Median (IQR)		
ICU stay	4.8 ± 0.38	4.6 ± 0.32	0.14	0.78
	3.5 (3–6)	4.0 (3–6)	(− 0.9 to 1.1)	
Hospital stay	30.8 ± 5.1	28.1 ± 3.0	2.6	0.66
	22 (17–34)	21 (18–34)	(− 9.2 to 14.5)	

*AF96h* atrial fibrillation within 96 h, *FAS* full analysis set, *PPS* per-protocol set, *AF24h* atrial fibrillation within 24 h, *ICU* intensive care unit, *CI* confidence interval, *SE* standard error, *IQR* interquartile range

*Fisher’s exact test

None of the secondary outcomes were significantly different between the two groups (Table 2). Details of all complications are shown in Table 3; the development of another complication in the same patient was counted as a separate complication (i.e., counted twice). Table 4 shows the time of AF onset. Intraoperative AF occurred in five patients, all of whom underwent electrical cardioversion that was ordered by the senior anesthesiologist. All returned to sinus rhythm. Six patients developed AF after the first postoperative day. Five of the six progressed well, with a natural return to sinus rhythm. One patient was deemed to have strong palpitations and was administered flecainide, after which sinus rhythm returned. All 11 patients who developed AF were discharged with sinus rhythm.

**Table 3:** Details of all complications in landiolol and placebo groups

	Clavien grade II complications		Clavien grade ≥ III (severe) complications	
Type of complication	Landiolol (***n*** = 28)	Placebo (***n*** = 28)	Landiolol (***n*** = 28)	Placebo (***n*** = 28)
All	4 (14.2)	7 (25.0)	7 (25.0)	7 (25.0)
Mortality	0 (0.0)	0 (0.0)	1 (3.6)	0 (0.0)
Gastric tube necrosis	1 (3.6)	0 (0.0)	2 (7.1)	0 (0.0)
Anastomotic stenosis	0 (0.0)	0 (0.0)	1 (3.6)	0 (0.0)
Anastomotic leakage	1 (3.6)	0 (0.0)	5 (17.9)	3 (10.7)
Pneumonia	0 (0.0)	3 (10.7)	0 (0.0)	0 (0.0)
Vocal cord paralysis	2 (7.1)	3 (10.7)	0 (0.0)	0 (0.0)
Supraventricular tachycardia	0 (0.0)	1 (3.6)	0 (0.0)	0 (0.0)
Sepsis	0 (0.0)	1 (3.6)	0 (0.0)	0 (0.0)
Pleural effusion	0 (0.0)	1 (3.6)	0 (0.0)	4 (14.3)
Intestinal ischemia	0 (0.0)	0 (0.0)	1 (3.6)	0 (0.0)
Intrathoracic bleeding	0 (0.0)	0 (0.0)	0 (0.0)	1 (3.6)

Data are expressed as number (%)

**Table 4:** Time of atrial fibrillation onset, starting from 9:00 am on the day of surgery

Parameter	Onset during surgery	Onset 24–48 h postop	Onset 72–96 h postop
All patients (*n* = 56)	5 (8.9)	4 (7.1)	2 (3.6)
Landiolol (*n* = 28)	3 (10.7)	3 (10.7)	0 (0.0)
Placebo (*n* = 28)	2 (7.1)	1 (3.6)	2 (7.1)

Data are expressed as number (%) of patients

There were no cases of atrial fibrillation onset at either 24 h or 48–72 h postoperatively

*postop* postoperatively

Some of the findings in this randomized trial were attributable to the pre-registration protocol. First, we added the AF24h outcomes with no pre-registration. Because landiolol is an ultrashort-acting drug, we later considered that the period during which the drug effect was exhibited was important. Second, for two patients, preoperative echocardiography could not be performed in the physiology laboratory, so their ejection fraction data are missing. However, the anesthesiologists visually confirmed a left ventricular ejection fraction of > 30% in these two patients immediately before the induction of anesthesia. Third, we performed a sensitivity analysis, adjusting for potential confounding factors resulting from the sample size shortage that was caused by early termination of the study.

## Discussion

**Fig. 2.**
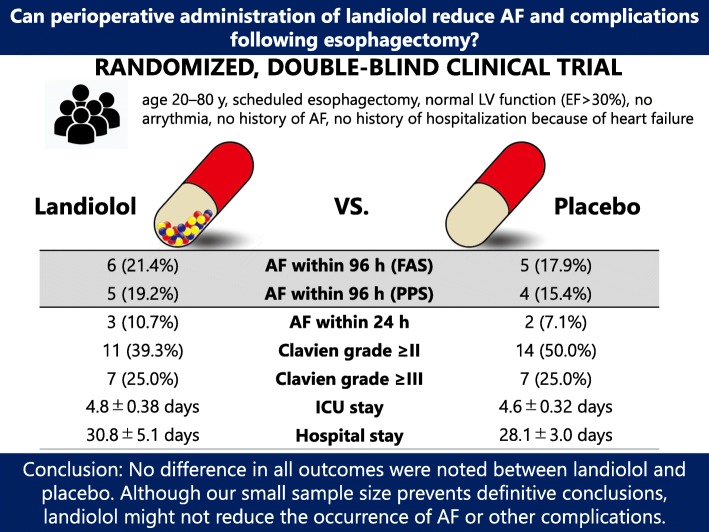
Graphical abstract. AF, atrial fibrillation; LV, left ventricular; FAS, full analysis set; PPS, per-protocol, set; ICU, intensive care unit

We hypothesized that landiolol administration can reduce the occurrence of AF and avoid complications during the perioperative period in patients undergoing esophagectomy. However, our results do not support this hypothesis: landiolol administration for 24 h from the start of surgery to the following day did not reduce the occurrence of AF within either 24 or 96 h. Additionally, landiolol was not associated with in-hospital morbidity or the duration of the ICU or hospital stay. In summary, although our sample size was small because of early study termination, landiolol failed to improve all outcomes during esophagectomy (Fig. 2, graphical abstract).

Our study showed that landiolol at 3 μg/kg/min administered for 24 h during and after esophagectomy did not reduce the occurrence of AF over a 4-day period, which is in contrast to the findings of two previously published randomized controlled trials. One of these trials showed that administration of landiolol at 3 μg/kg/min for 72 h after esophagectomy reduced the occurrence of AF for 7 days postoperatively (i.e., the incidence of AF was 10% with landiolol vs. 30% with placebo, with 50 patients in each group) [11]. The other study showed that administration of landiolol at 5 μg/kg/min for 24 h during and after esophagectomy reduced the occurrence of AF for 2 days postoperatively (the incidence of AF was 5.3% with landiolol vs. 25.0% with placebo, with 20 patients in each group) [12]. Another study involving cardiac procedures showed that administration of landiolol at 0.5 μg/kg/min for 3 days reduced re-occurrence of AF (the incidence of AF was 16% with landiolol vs. 48% with placebo, with 25 patients in each group) [17]. Because the prophylactic efficacy of landiolol for preventing AF during cardiac surgery has been established [6–10], our results were unexpected. The general belief is that that landiolol has an AF-preventive effect, but our results showed different trends. We believe that it is important to publish the present study, in which an appropriate methodology was used, to avoid publication bias. Moreover, because our allocation concealment and blinding were more robust than those in previous studies, our results should be considered reliable. Further randomized trials of patients undergoing esophagectomy are expected to evaluate the contribution of parameters such as the administration period, dosage, and timing.

In the present study, landiolol did not reduce the frequency of Clavien–Dindo complications in patients undergoing esophagectomy. In this patient population, AF may occur as a prodromal symptom of complications because these two entities are closely related [18–20]. An earlier randomized trial showed that landiolol reduced the proportion of Clavien–Dindo complications, although the protocol was switched to an open-label design after AF was diagnosed [11]. The authors explained that landiolol reduced the incidence of complications because of its anti-inflammatory effect, as shown by declining postoperative interleukin-6 levels compared with the placebo group. Based on these findings, however, we suggest that the anti-inflammatory effect of landiolol is not yet established and that there is a lack of evidence that landiolol can reduce complications following esophagectomy.

In the present study, landiolol did not affect the duration of the ICU and hospital stays. Several studies have shown that the occurrence of perioperative AF leads to increased lengths of ICU and hospital stays following noncardiac surgery [21, 22]. A previous randomized trial demonstrated that landiolol did not shorten the duration of hospitalization, although it did prevent AF during esophagectomy [12]. However, both the previous randomized trial and the present trial examined the hospitalization period as a secondary endpoint because of the insufficient sample size. In a recent study, sustained AF led to a high mortality rate and longer length of ICU stay in noncardiac patients in the ICU [23]. These post-esophagectomy outcomes are also important.

The present study has several limitations. First, this study was terminated early, before the planned number of patients could be enrolled. Although randomized controlled trials that stop early tend to overestimate treatment effects [24], our results may be attributable to low detection power because of the small sample size. We do not believe that the sample size was too small compared with previous similar studies, but it is an important limitation in this study. Second, we did not collect hemodynamic data (e.g., mixed venous oxygen saturation, lactate concentration), disturbances in electrolytes (e.g., potassium, magnesium), or management of vasopressors, inotropes, or anesthetics. The double-blind randomized design allowed for control of unmeasured or unknown confounding factors, and we additionally performed the sensitivity analysis to adjust for the background differences between the groups. Low-dose landiolol has been shown to regulate the heart rate with few adverse effects because of its high β1 selectivity and limited negative inotropic effects [6]. Although detailed hemodynamic data were not collected in the present study, perioperative management was not difficult in any patients in either group. Third, this study was performed in a single center in Japan. The generalizability (external validity) may not be established as in other randomized controlled trials, and this is an issue for the future. Finally, this randomized trial excluded patients with a history of AF or low heart function, which may indicate a high risk of AF occurrence. Because these patients are more likely to benefit from landiolol, it may be a notable patient background factor in future studies.

## Conclusion

In conclusion, administration of landiolol for 24 h did not reduce the occurrence of AF or other complications compared with placebo, although our small sample size prevents definitive conclusions. Little evidence is available to support the use of landiolol during esophagectomy, meaning that a prospective trial to investigate the clinical effects of this therapeutic approach is warranted.

## Supplementary information

**Additional File 1.** Hemodynamic data at each point in the study.

**Additional File 2.** Sensitivity analysis for the primary outcome.

## Data Availability

Not applicable.

## Abbreviations

AF: Atrial fibrillation
CONSORT: Consolidated Standards of Reporting Trials
ICU: Intensive care unit
TCI: Target-controlled infusion
FAS: Full analysis set
PPS: Per-protocol set
CI: Confidence interval
ORs: Odds ratios
