# Supplementary material for: Landiolol hydrochloride for prevention of atrial fibrillation during esophagectomy: a randomized controlled trial
Source: JA Clin Rep. 2020 May 11;6:34. doi: 10.1186/s40981-020-00338-3 (PMC7214548; doi:10.1186/s40981-020-00338-3)
Supplement: Supplementary file 1 — Additional File 1. Hemodynamic data at each point in the study. [file 40981_2020_338_MOESM1_ESM.docx]

| **Timing** | **Hemodynamic data** | **Landiolol** | **Placebo** | **P value*** |
| --- | --- | --- | --- | --- |
| Pre-op | Heart rate | 80 (74–85) | 83 (80–90) | 0.12 |
|  | Systolic BP | 136 (130–146) | 139 (125–142) | 0.7 |
|  | Diastolic BP | 70 (70–80) | 76 (70–80) | 0.41 |
| Intra-op | Heart rate | 78 (70–83) | 79 (70–90) | 0.3 |
|  | Systolic BP | 102 (100–110) | 102 (100–110) | 0.87 |
|  | Diastolic BP | 51 (50–62) | 59 (52–60) | 0.28 |
| ICU admission | Heart rate | 79 (75–84) | 81 (77–89) | 0.21 |
|  | Systolic BP | 111 (105–129) | 119 (107–129) | 0.59 |
|  | Diastolic BP | 71 (64–77) | 76 (71–80) | 0.034 |
| Day after surgery | Heart rate | 80 (69–85) | 81 (79–90) | 0.065 |
|  | Systolic BP | 118 (100–122) | 120 (110–130) | 0.18 |
|  | Diastolic BP | 60 (51–70) | 70 (60–76) | 0.026 |
| ICU exit | Heart rate | 85 (80–100) | 87 (80–100) | 0.93 |
|  | Systolic BP | 129 (120–132) | 122 (112–138) | 0.47 |
|  | Diastolic BP | 80 (80–85) | 80 (80–90) | 0.77 |

**Additional File 1. Hemodynamic data at each point in the study**

Data are presented as median (interquartile range).

Heart rate is presented in beats/min; BP is presented in mmHg.

*Probability > chi-square

Pre-op, preoperative; Intra-op, intraoperative; ICU, intensive care unit; BP, blood pressure
