# Supplementary material for: Landiolol hydrochloride for prevention of atrial fibrillation during esophagectomy: a randomized controlled trial
Source: JA Clin Rep. 2020 May 11;6:34. doi: 10.1186/s40981-020-00338-3 (PMC7214548; doi:10.1186/s40981-020-00338-3)
Supplement: Supplementary file 2 — Additional File 2. Sensitivity analysis for the primary outcome. [file 40981_2020_338_MOESM2_ESM.docx]

**Additional File 2.** **Sensitivity analysis for the primary outcome**

| **Measurements** | **Estimate value** | **Standard error** | **P value*** |
| --- | --- | --- | --- |
| Segment | −8.18 | 8.35 | 0.33 |
| Allocation (landiolol or placebo) | 0 | 0 | 1 |
| Age (years) | 0.049 | 0.048 | 0.30 |
| Sex (male or female) | 0.19 | 0.88 | 0.83 |
| Preoperative ejection fraction (%) | −0.086 | 0.11 | 0.42 |
| Diabetes mellitus (yes or no) | −2.34 | 1.63 | 0.15 |
| BMI (kg/m^2^) | 0 | 0 | 1 |
| Preoperative eGFR (mL/min/1.73 m^2^) | 0.12 | 0.036 | 0.0007 |
| Hypertension (yes or no) | 0 | 0 | 1 |
| Hyperlipidemia (yes or no) | 1.40 | 1.32 | 0.29 |
| FEV_1.0%_ (%) | 0 | 0 | 1 |
| Surgery time (min) | 0 | 0 | 1 |
| Anesthesia method (TIVA or inhalation) | 1.33 | 0.86 | 0.12 |
| Inotropic agents (dopamine or dobutamine) within 96 h (yes or no) | −0.98 | 0.79 | 0.21 |
| Vasopressor agents (norepinephrine or epinephrine) within 96 h (yes or no) | 0 | 0 | 1 |
| Abnormal potassium concentration within 96 h (yes or no) | −0.17 | 0.93 | 0.86 |
| Abnormal sodium concentration within 96 h (yes or no) | 2.56 | 0.83 | 0.002 |
| Sedative agents (propofol or dexmedetomidine or midazolam) in the ICU (yes or no) | 1.03 | 1.48 | 0.49 |

*Probability > chi-square

BMI, body mass index; eGFR, estimated glomerular filtration rate; FEV_1.0%_, forced expiratory volume % in 1 s; TIVA, total intravenous anesthesia; ICU, intensive care unit
